# ORMDL3 and Asthma: Linking Sphingolipid Regulation to Altered T Cell Function

**DOI:** 10.3389/fimmu.2020.597945

**Published:** 2020-11-30

**Authors:** Christopher R. Luthers, Teresa M. Dunn, Andrew L. Snow

**Affiliations:** ^1^ Department of Pharmacology and Molecular Therapeutics, Uniformed Services University of the Health Sciences, Bethesda, MD, United States; ^2^ Department of Biochemistry, Uniformed Services University of the Health Sciences, Bethesda, MD, United States

**Keywords:** asthma, orosomucoid like sphingolipid biosynthesis regulator 3, T cell, sphingolipids, allergic inflammation

## Abstract

*Orosomucoid like 3 (ORMDL3)* encodes an ER-resident transmembrane protein that regulates the activity of serine palmitoyltransferase (SPT), the first and rate-limiting enzyme for sphingolipid biosynthesis in cells. A decade ago, several genome wide association studies revealed single nucleotide polymorphisms associated with increased ORMDL3 protein expression and susceptibility to allergic asthma. Since that time, numerous studies have investigated how altered ORMDL3 expression might predispose to asthma and other autoimmune/inflammatory diseases. In this brief review, we focus on growing evidence suggesting that heightened ORMDL3 expression specifically in CD4^+^ T lymphocytes, the central orchestrators of adaptive immunity, constitutes a major underlying mechanism of asthma pathogenesis by skewing their differentiation and function. Furthermore, we explore how sphingolipid modulation in T cells might be responsible for these effects, and how further studies may interrogate this intriguing hypothesis.

## Introduction

Asthma is a chronic lung disease associated with narrowing of airways, bronchial hyperreactivity, and increased mucus production. This disease affects an average of 330 million individuals worldwide and 24.7 million people across the US, including 439,000 hospitalizations and 3400 asthma-related deaths (1). Asthma is influenced by both environmental and genetic factors, with variants in many genes being strongly associated with increased asthma susceptibility and/or pathophysiology. Importantly, asthma is now recognized as a clinically heterogeneous disease associated with many different genetic alterations as well as phenotypic outcomes for the same disease. Previous studies solely focused on phenotypes and grouped individuals with symptoms into two categories of allergic vs. non-allergic asthma. Recent studies have, however, focused more heavily on pathophysiological symptoms of patients and grouped patients into different endotypes (2).

Several genome wide association studies (GWAS) interrogating the strong genetic component of asthma have linked SNPs in the non-coding chromosomal regions of 17q12-21 with both childhood and adult asthma in humans (3–5). Specific endotype associations remain unclear, although most data point to a stronger association with childhood asthma, which is often but not always allergic (5, 6). These SNPs affect the expression of several genes, most notably *ORMDL3*. ORMDL3 and its isoforms, ORMDL1 and ORMDL2, are part of a family of highly conserved transmembrane proteins residing in the endoplasmic reticulum (ER). ORMDL3 function has been previously linked with ER calcium homeostasis (7, 8) inflammatory responses (9, 10), and the ER stress response (11–13). In 2010, two major studies showed that the yeast homologs of the ORMDLs, the ORM proteins, are negative regulators of the committed and rate-limiting enzyme of sphingolipid biosynthesis, serine palmitoyltransferase (SPT) (14, 15). This function was later corroborated for mammalian ORMDL3 (16–20); ORMDL3 protein is highly conserved between mouse and humans (>96% identical), with similar patterns of tissue-specific expression. Sphingolipids are a family of lipids involved in membrane rigidity and structure and they also confer cell identity and serve as receptors for multiple pathogens. Several sphingolipid species such as S1P and ceramide are also known to play key roles in immune signaling (21, 22). Furthermore, sphingolipid dysregulation has been implicated in several respiratory diseases, such as COPD and cystic fibrosis (23).

Various functions assigned to ORMDL3 may impact asthma pathogenesis by altering the physiology of several cell types. Indeed, global Ormdl3 overexpression led to increased pathology and airway hyper-reactivity at baseline and in an ovalbumin-induced asthma mouse model (24), although a separate study using Ormdl3 transgenic mice failed to show any exacerbation of allergen-induced experimental asthma (25). In humans, a recent study assessed expression of ORMDL3 mRNA in various tissues from patients harboring the 17q12–21 risk SNPs. Strikingly, the most dramatic changes in ORMDL3 expression were noted in immune cells, particularly in CD4^+^ T lymphocytes, which showed a 3 fold increase in ORMDL3 mRNA (7). Furthermore, enhanced ORMDL3 expression was shown to have functional consequences, including reduced expression of interleukin-2 from T cells (IL-2). These findings imply a direct intrinsic role for increased ORMDL3 expression in human T cells to drive heightened asthma pathophysiology. Indeed, transgenic ORMDL3 overexpressing mice also exhibited increased Th2 responses and airway hyperresponsiveness in response to allergen challenge (24).

The contribution of ORMDL3 overexpression has also been studied in non-immune cells such as airway smooth muscle (ASM) and bronchial endothelial (BEC) cell types. Although hyperactive ASM and BEC in the mucosal epithelium are unquestionably a significant component of asthma pathophysiology, it remains unclear as to whether abnormal ORMDL3 expression in these tissues exacerbates disease, or whether the association of elevated ORMDL3 with asthma might be mediated primarily in immune cells like CD4^+^ T cells. Certainly, the importance of the underlying immune response and its correlation to allergic asthma cannot be overstated. In this review, we explore how intrinsic ORMDL3 overexpression in human T cells may contribute to asthma pathogenesis. By re-examining previous studies in immune and non-immune cells from humans, mouse models and even lower organisms (e.g. yeast), we attempt to elucidate potential mechanisms by which ORMDL3 overexpression in CD4^+^ T cells may connect to enhanced pathophysiology of asthma in patients carrying the 17q12-21 risk SNPs.

## ORMDL3, SPT, and Sphingolipid Regulation

**Figure 1:**
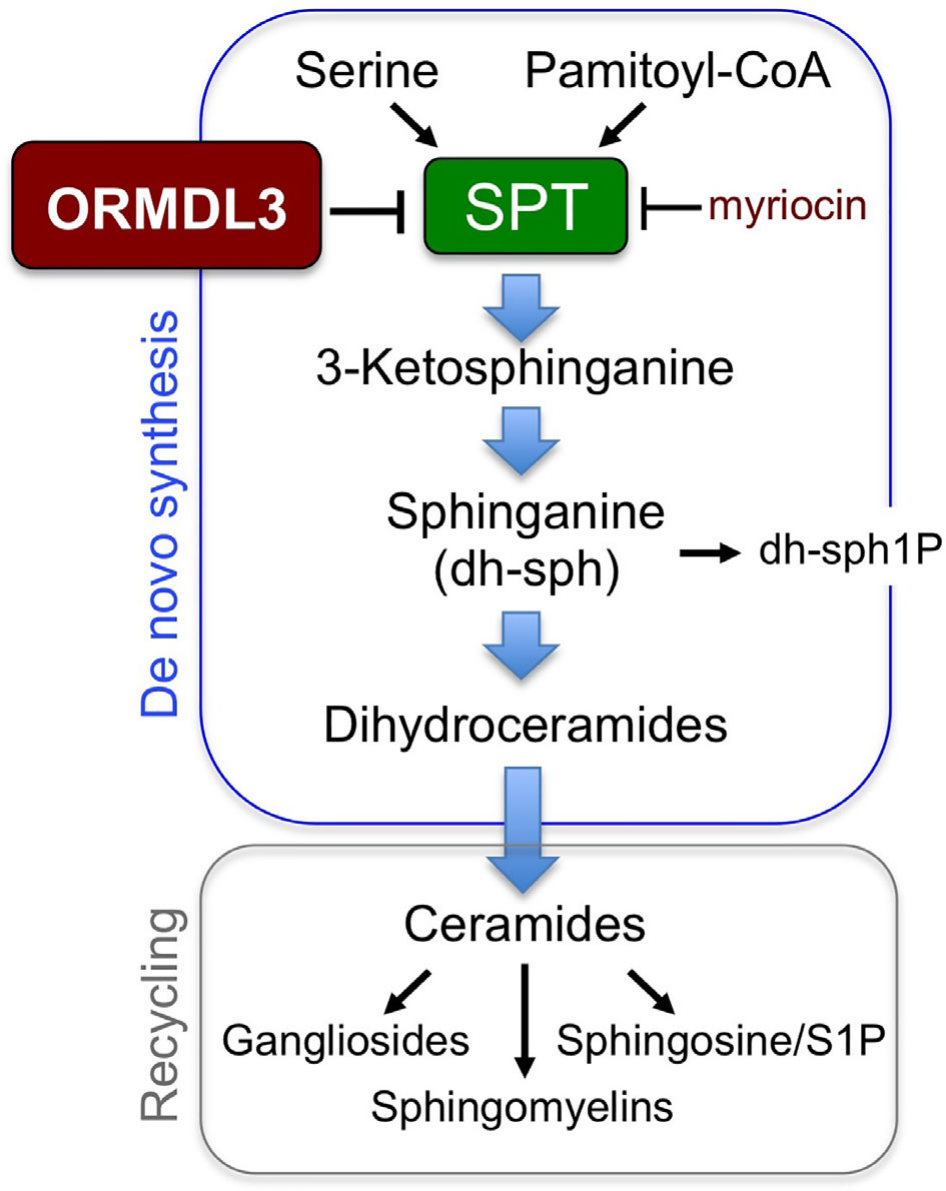
Sphingolipid synthesis pathways. ORMDL3 regulates the activity of serine palmitoyl-CoA transferase (SPT), the first and rate-limiting enzyme for sphingolipid biosynthesis.

As previously stated, the major molecular function of ORMDL3 is to negatively regulate the rate limiting enzyme in sphingolipid biosynthesis, SPT, influencing all species of sphingolipids downstream (**Figure 1**). With perhaps one exception (26), ORMDL3-dependent SPT regulation has been demonstrated in many cell types over several different studies (14–20), presenting a reasonable hypothesis that sphingolipid dysregulation could contribute to asthma pathophysiology in patients with the 17q12–21 risk SNPs. This hypothesis is supported by a study in which Lindsley et al. administered intratracheal myriocin in a house dust mite (HDM) sensitization model in mice, and measured cytokine secretion, airway hyperresponsiveness, and bronchial lavage cytology (27). Myriocin is a potent pharmacological inhibitor of SPT, making it an effective tool to mimic effects that ORMDL3 overexpression would have on SPT regulation and sphingolipid biosynthesis. Mice sensitized with HDM plus myriocin demonstrated a 63% increase in airway hyperresponsiveness (AHR) relative to those sensitized with HDM alone (27). Increased AHR correlated with elevated Th2 cell counts and increased expression of Th2 cytokines (IL-5, IL-13) in mice that were treated with HDM/myriocin (27).

To further support this hypothesis, Worgall et al. utilized a genetic approach to confirm that dysregulation of sphingolipid homeostasis can exacerbate asthma phenotypes, employing heterozygous SPT knockout mice in comparison to myriocin treatment (28). In this study, SPT-/+ mice phenocopied administration of myriocin in WT mice, exhibiting decreased production of several different sphingolipid species including sphinganine, sphingosine-1-phosphate, and ceramide in these mice (28). Similar to myriocin treatment of WT mice, SPT-/+ mice showed increased airway resistance and contractile response of lungs in response to methacholine challenge (28).

Although these two studies provide ample evidence of a mechanistic link between sphingolipid dysregulation and exacerbation of allergic asthma, several unanswered questions remain. It remains unclear whether ORMDL3 overexpression in mice mimics all phenotypes observed with either myriocin treatment or in SPT-/+ mice, including altered sphingolipid levels, Th2 cytokines and IgE production, and enhanced functional readouts such as AHR. Indeed, aforementioned studies of global Ormdl3 transgenic mice have yielded contradictory results (24, 25). The recent study by Debeuf et al. clearly demonstrated reduced versus increased sphingolipid levels in Ormdl3 transgenic versus knockout mice, respectively, in contrast to their earlier report claiming ORMDL3 had no influence on SPT activity (26). Nevertheless, key asthma features remained unaltered relative to wild-type mice in their allergen asthma models (25), differing from earlier findings from Miller et al (24). These mixed results arguably emphasize a separate unanswered question as to which cells exhibiting heightened ORMDL3 expression may be most relevant in driving asthma pathology, and whether functional abnormalities are primarily driven by altered sphingolipid biosynthesis in specific cells. This interesting avenue is well worth pursuing with clinical relevance to patients with 17q12–21 risk SNPs, necessitating the development of mouse models in which ORMDL3 expression is manipulated in distinct cell types, including CD4^+^ T cells. Recent studies employing conditional knockouts of ORMDL3 in airway epithelium have yielded mixed results in murine asthma models (3, 29, 30), demanding further investigation of ORMDL3 in additional tissues.

## ORMDL3 Overexpression, SERCA2B, and ER Stress

Asthma is a heterogeneous disease with complex clinical expressions. Recent evidence links ER stress and the unfolded protein response (UPR) to asthma exacerbation (31). ER stress-induced UPR can lead to immune dysregulation and endogenous inflammatory responses. Aside from SPT regulation, ORMDL3 is also reported to regulate the sarcoendoplasmic reticulum (SR) calcium transport ATPase (SERCA) pump, which transports calcium ions from the cytoplasm into the SR (8). This process is vital for maintaining homeostatic levels of calcium ions inside the ER, and any dysregulation of ER calcium levels can result in increased ER stress, UPR, and exacerbation of asthma pathogenesis. Changes in ORMDL3-dependent regulation of the SERCA pump could elicit ER stress and UPR in multiple cell types, including CD4^+^ T cells, contributing to increased incidence and/or severity of asthma in patients with 17q12–21 risk SNPs. Although it has been reported that ORMDL3 associates directly with SERCA2B (5), the possibility that perturbations in Ca^2+^ homeostasis are secondary to altered regulation of sphingolipid levels deserves further investigation. Regardless of whether the effects are direct or mediated through altered sphingolipids, there is clear evidence that ORMDL3 expression modulates Ca^2+^ homeostasis.

Vicente and colleagues showed that ORMDL3 regulates the SERCA pump in human HEK293 and Jurkat T cells, leading to decreased calcium ion levels in the ER (8). Overexpression of ORMDL3 also led to a decrease in ER-mediated calcium signalling, with a concomitant increase in resting calcium levels in the cytosol (8). This effect was reversed by overexpressing SERCA. ORMDL3 overexpression also led to activation of the UPR; conversely, knockdown of ORMDL3 increased calcium release from the ER and a diminished UPR (8). Hence relative ORMDL3 expression controls ER stress and UPR *via* SERCA regulation.

A novel study by Papp et al. discovered a dynamic role for SERCA expression upon activation of T lymphocytes. In this study, it was shown that upon activation of human T cell lines using PMA and ionomycin, expression of specific SERCA isoenzymes was dramatically altered within 96 h (32). These changes in SERCA isoform expression were concurrent with enhanced expression of both IL-2 and the IL-2 receptor (32). Furthermore, the calcineurin inhibitor cyclosporine reversed the effects on SERCA isoform expression, concurrent with a stark decrease in IL-2 expression (32). The Broide group also showed that human ORMDL3 overexpression results in increased SERCA2B levels in murine airway smooth muscle cells (33). Together, these studies underscore an essential role for the SERCA pump and ER-dependent calcium dynamics during T cell activation, growth and proliferation. From these findings, we might hypothesize that in patients carrying 17q12–21 asthma risk SNPs, dynamic changes in the expression and action of key ER SERCA pumps in response to T cell receptor (TCR) activation would be hindered by higher ORMDL3 expression in CD4^+^ T cells. This would ostensibly result in reduced calcium influx through SERCA pumps to replenish ER stores and increased cytosolic calcium concentrations in resting T cells, altering a key rheostat of early T cell signaling (34). This dysregulation might also result in changes to ER stress, UPR activation, and altered T cell differentiation and function, as suggested by recent studies (35, 36). In fact, inhibition of the specific UPR activator IRE1α can reduce the expression of Th2 cytokines in murine T cells (36, 37).

If this novel hypothesis presents a potential alternative avenue for exploring CD4^+^ T cell-dependent asthma pathogenesis in patients with 17q12-21 risk SNPs, further detailed characterization of this potential phenotype is required. Studies involving genetic ablation or pharmacologic inhibition of SERCA pumps in CD4^+^ T cells could be employed to determine how SERCA-dependent dysregulation of ER calcium stores alters ER stress, UPR and T cell function, and whether manipulation of ORMDL3 levels change these effects in a SERCA-dependent manner.

## ORMDL3 Overexpression and Lymphocyte Activation

Several studies have now linked ORMDL3 expression with altered early and late signaling events in lymphocyte activation. For example, ORMDL3 modulates store operated calcium entry (SOCE), and thus activation of human Jurkat CD4^+^ T cells (38). Specifically, ORMDL3 overexpression led to increased basal cytosolic calcium levels and decreased extracellular calcium influx upon TCR stimulation. Conversely, siRNA-mediated knockdown of ORMDL3 increased extracellular calcium influx; the opposite effect seen with SERCA silencing. Consistent with these data, ORMDL3 overexpression led to inhibition of calcium release-activated currents (I(CRAC)), reduced SOCE, and decreased nuclear translocation of nuclear factor of activated T-cells (NFAT), a key transcription factor required for sustained activation and proliferation of effector CD4^+^ T cells (38). In turn, these signaling defects resulted in decreased IL-2 production after T cell activation. Again, ORMDL3 knockdown produced the opposite effects (38). Hence this study established a novel role for ORMDL3 in early calcium signalling and IL-2 production following TCR activation.

In a more physiological context, we know primary CD4^+^ T cells from humans harboring 17q12–21 asthma risk SNPs display ~3-fold overexpression of ORMDL3 (7). We suspect this results in higher basal calcium levels in the cytosol for naive CD4^+^ T cells. However, antigen/allergen recognition *via* TCR ligation will produce suboptimal SOCE/calcium influx, reduced nuclear translocation of NFAT, and poor IL-2 production. Consequently, diminished IL-2 levels could contribute to impaired growth of conventional CD4^+^ T cells, skewed Th2 differentiation, and/or defective maintenance of regulatory T cells, which are highly dependent on paracrine IL-2 for survival and suppressive function (39). In the end, we hypothesize that skewed CD4^+^ T cell differentiation in the context of elevated ORMDL3 likely culminates in chronic inflammation associated with heightened disease pathogenesis in asthma patients carrying the 17q12–21 risk SNPs (40).

More experiments are needed to test the validity of this hypothesis. First, direct manipulation of ORMDL3 expression should be interrogated in both primary human and murine T cells to provide more physiological relevance, including *in vivo* experiments using aforementioned mouse models. If effects on T cell activation parameters are consistent with previous findings in Jurkat T cells, further experiments would be required to demonstrate that dysregulation of Ca^2+^ signaling in T cells is directly responsible for driving asthma pathophysiology, and whether these effects reflect altered sphingolipid homeostasis. Such studies will shed further light on the molecular and cellular mechanisms underpinning increased asthma pathogenesis in patients with asthma risk SNPs that enhance ORMDL3 expression in T cells.

## ORMDL3 Overexpression, CD4^+^ T Cell Differentiation, and Cytokines

Enhanced production of Th2 effector CD4^+^ T cells and their signature cytokines (e.g. IL-4, IL-5, IL-13) are a major contributor to allergic asthma in humans. Hence, one could hypothesize that patients with 17q12-21 asthma risk SNPs correlating with ORMDL3 overexpression may display increased Th2 CD4^+^ T-cells and associated cytokine profiles. Indeed, this was suggested by a study by Kabesch et al. that confirmed patients carrying the 17q12–21 SNPs show an increased incidence of asthma, with a substantially stronger association noted for atopic versus non-atopic asthma (41). Importantly, peripheral blood mononuclear cells (PBMCs) from patients homozygous for the asthma risk SNPs showed increased ORMDL3 mRNA expression and significantly elevated IL-4 and IL-13 production in response to mitogenic and allergen stimuli ex vivo, compared to non-risk SNP carriers. These effects were diminished in heterozygous SNP carriers, but were still higher than in PBMCs isolated from patients homozygous for the non-risk allele (41). This novel study was the first to posit the intriguing theory that ORMDL3 overexpression might skew CD4^+^ T cell differentiation toward a Th2 imbalance, contributing and priming the immune response toward allergic asthma.

However, a more recent study from the same group suggests an alternative theory. Shaub et al. studied cord blood leukocytes from a cohort of 200 17q21 risk SNP carriers, reporting higher expression of locus-associated ORMDL3 and gasdermin B (GSDMA) and slightly increased IL-17 production upon allergen stimulation (42). These findings illuminate a possible association of asthma risk SNPs and ORMDL3 overexpression with IL-17 production early in life, affecting early immune maturation in asthmatic patients. Newer endotype models of asthma implicate a role for Th17 cells in contributing to distinct immunopathology independent of Th2-mediated responses. Several groups have reported a role for Th17 cells in the development of asthmatic endotypes associated with enhanced IL-17–dependent recruitment of neutrophils to the lungs (43, 44). Clinically, this subset of patients responds poorly to steroid treatment, which is directly attributed to neutrophilic inflammation of the airways (44). Furthermore, increased expression of IL-17 has been correlated with severe asthma in humans, with increased neutrophilic infiltrates evident in mucus (45). Intriguingly, a separate study reported that patients homozygous for a SNP that introduces a loss of function mutation in IL-17F protein (H161R) were protected from asthma (46). This inverse correlation was attributable to the H161R protein functioning as a natural IL-17F antagonist, implying a critical role for IL-17F in asthma pathogenesis (46). Any epistatic relationship between this IL-17F SNP and the 17q21 asthma-risk SNPs remains to be determined.

Immune homeostasis demands the careful regulation CD4^+^ T cell differentiation and effector functions essential for proper clearance of specific pathogens without triggering overt tissue damage. CD4^+^ T cell dysregulation or imbalance can lead to autoimmunity, asthma, or prolonged infection. In asthma, a hyperactive immune response to allergens can drive airway hyperreactivity and bronchial constriction. Although this response is classically associated with an underlying Th2-mediated inflammatory response, the development of asthma-like symptoms can also be linked to increased production of Th17 cells and secretion of IL-17. This will result in increased neutrophil recruitment and neutrophilic inflammation in the airways, resulting in a distinct asthma endotype. Remarkably, the aforementioned studies link 17q21 risk SNPs and increased ORMDL3 levels in human asthmatic patient CD4^+^ T cells to atypical differentiation of *both* Th2 or Th17 cells. Thus, it may be that enhanced ORMDL3-dependent modulation of sphingolipid synthesis in CD4^+^ T cells results in improper allergic or neutrophilic inflammatory responses across the clinical spectrum of asthma phenotypes.

While plausible, several pivotal studies are needed to explore this potential hypothesis. First, a causative relationship must be established between increased ORMDL3 expression and differential Th2 or Th17 skewing in CD4^+^ T cells, by altering ORMDL3 expression experimentally. Changing ORMDL3 expression is expected to cause concomitant fluctuations in sphingolipid homeostasis. In the context of such experiments, the introduction of mutations that disrupt interaction between ORMDL3 and SPT might be expected to abrogate effects on both sphingolipid synthesis and T cell differentiation. If sphingolipid perturbations potentially underlie altered T cell skewing, effects associated with WT ORMDL3 overexpression might be mimicked *in vitro* by culturing CD4^+^ T cells in the presence of myriocin or other inhibitors of downstream enzymes in the sphingolipid synthesis pathway under Th2 or Th17 polarizing conditions. Indeed, our preliminary studies in Jurkat and primary human T cells indicate clear links between varied ORMDL3 expression and changes in TCR signaling and Th2 skewing that are reflective of changes in sphingolipid regulation (C. Luthers, data not shown).

## ORMDL3 and T Cell Metabolic Fitness

Activated T cells must be able to rapidly reprogram their cellular metabolism in order to mount an effective immune response to foreign pathogens. Dysregulation of this process can result in failure to control infection or immunopathology (47). As new links emerge connecting immunometabolism to abnormal immune responses like asthma, a recent study suggests sphingolipid synthesis may be an important factor in T cell metabolic fitness. T cell responses in human patients with loss-of-function (LOF) mutations in the serine palmitoyltransferase subunit SPTLC2, one of three subunits comprising the rate limiting holoenzyme for sphingolipid biosynthesis, SPT were investigated. These missense mutations reduce SPT catalytic activity and shift its substrate specificity, resulting in the accumulation of neurotoxic lipid species that ultimately cause Hereditary Sensory Neuropathy type I (HSAN-I). Aside from severe neurologic complications including loss of nociception, these patients also suffer from recurrent infections.

Cui and colleagues asked how HSAN-I associated SPTLC2 mutations might affect human and murine CD8^+^ T cell function. Surprisingly, CD8+ T cells from HSAN-I patients showed attenuated proliferation, survival and cytokine production upon *in vitro* stimulation, suggesting a novel link between sphingolipid regulation and T cell effector function. Indeed, antigen stimulation resulted in a significant upregulation of SPTLC2 expression in normal T cells. To study this link further, conditional knockout mice lacking Sptlc2 in T cells were challenged with lymphocytic choriomeningitis virus (LCMV) infection. The normally robust CD8^+^ effector T cell response to LCMV was severely impaired with SPTLC2-deficiency due to decreased metabolic fitness and increased cell death, attributed to prolonged mTORC1 activation and increased ER stress. Remarkably, these defects in SPTLC2-deficient murine T cells and HSAN-I patient T cells were rescued by supplementation of sphingolipids in culture (48).

This study is the first to suggest that deranged sphingolipid biosynthesis can contribute to T cell metabolic defects. ORMDL3 overexpression might be contributing to similar defects as the LOF mutations in SPT subunits that attenuate sphingolipid synthesis. As metabolic reprogramming is also intimately linked to CD4^+^ T cell differentiation and effector function (49), this link is worth exploring further in the context of asthma. Reduced IL-2 production and fate skewing, as noted in human T cells homozygous for 17q12–21 asthma risk SNPs and elevated ORMDL3 expression, may result from reductions in sphingolipid generation that hinder the shift to anabolic metabolism and macromolecule biosynthesis required during clonal T cell expansion and Th1 effector differentiation (49).

## Discussion

**Figure 2:**
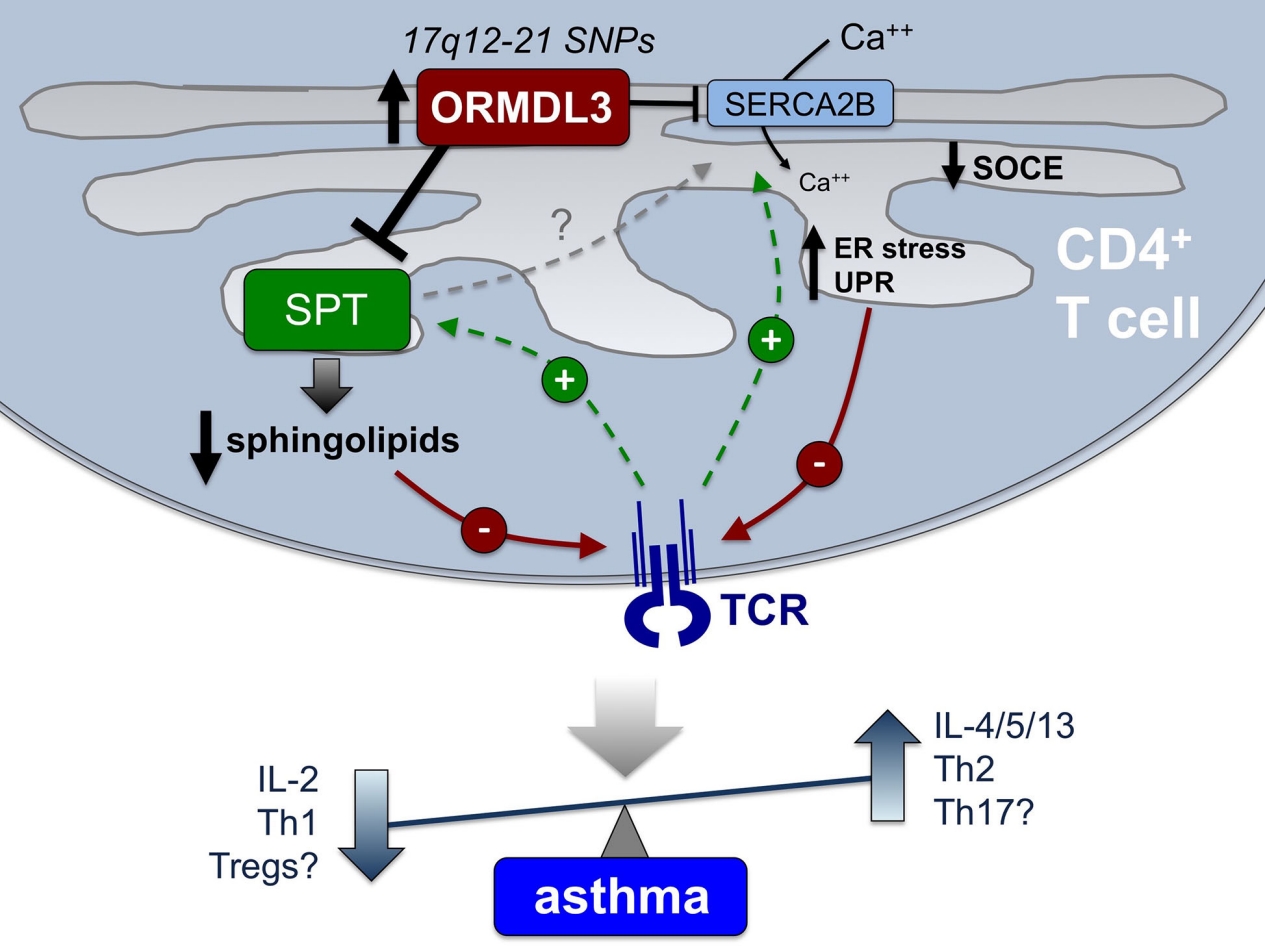
Proposed mechanisms connecting elevated ORMDL3 expression in CD4+ T cells to asthma pathogenesis. In CD4^+^ T cells from those harboring 17q12-21 asthma risk SNPs, elevated ORMDL3 expression is thought to modulate the activity of both SPT and SERCA2B, both of which are normally enhanced after TCR stimulation (green lines). Consequently, we hypothesize that TCR signaling is diminished (red lines) *via* reduced/altered sphingolipid synthesis, as well as defective SOCE and reduced TCR-induced Ca^++^ flux, and potential induction of ER stress and UPR. Attenuated TCR signaling and downstream T cell activation is known to result in reduced IL-2 production and skewing toward a Th2 phenotype (i.e. elevated IL-4, IL-5, and IL-13 secretion), highlighting a potential mechanistic association with allergic asthma pathogenesis that remains to be definitively shown. Other outcomes (reduced Tregs, enhanced Th17 cells) may also contribute to inflammation and non-atopic asthma in certain patients. Based on findings to date, we posit that altered sphingolipid synthesis *via* SPT regulation is the major driver of these changes in T cell differentiation and function, which may also indirectly influence calcium homeostasis and ER stress *via* SERCA2B.

Asthma pathogenesis likely involves synergistic dysregulation of both parenchymal cells (e.g. bronchial epithelium, airway smooth muscle cells) and resident or infiltrating immune cells. This review has focused on the role of human CD4^+^ T cells in exacerbating asthma phenotypes in the context of 17q12–21 asthma risk SNPs associated with heightened ORMDL3 expression. Several GWAS studies have identified the noncoding regions of SNPs in the 17q12–21 chromosomal region to be strongly linked to asthma in ethnically diverse populations. Furthermore, studies have shown that these patients have increased expression of several proteins, most notably ORMDL3. Indeed, ORMDL3 is particularly highly expressed in CD4^+^ T cells from these patients, correlating with reduced IL-2 production and potential Th2/Th17 skewing. Although various cellular functions of ORMDL3 have been characterized, its precise mechanistic role in altering CD4^+^ T cell function and enhancing asthma susceptibility has not been resolved. In this review, we have attempted to provide a roadmap for further defining these mechanisms, summarized in **Figure 2**.

It is also noteworthy that a multitude of studies have directly linked ORMDL3 overexpression with other human inflammatory disorders. Large GWAS studies have identified a significant association between ORMDL3 expression and inflammatory bowel diseases (IBD) including Crohn’s and ulcerative colitis (50, 51). ORMDL3 is also implicated as a causal gene of rheumatoid arthritis, an autoimmune disorder characterized by chronic inflammation of the joints (52). Intriguingly, chronic ER stress in gut epithelium and synovial tissues has been implicated in both IBD and RA, respectively (53, 54). ORMDL3-mediated ER stress and UPR may exacerbate proinflammatory cytokine production and tissue inflammation associated with these autoimmune disorders. In contrast, Xiao et al. found that children with type 1 diabetes had significantly lower ORMDL3 expression in peripheral blood leukocytes relative to healthy children (55). They further implicated ORMDL3 in promoting islet beta cell proliferation by activating transcription of ATF6, a major UPR protein (55). The UPR triggers cleavage of membrane ATF6, releasing its cytoplasmic domain for subsequent nuclear translocation and transactivation of chaperone genes for resolving ER stress (56). Collectively, these findings suggest a plausible mechanism connecting changes in ORMDL3 expression to the UPR, ER stress, and inflammation that warrants more exploration in the context of asthma. Indeed, new treatments to reduce ORMDL3 expression in the lungs have yielded promising results in ameliorating airway inflammation in mice, emphasizing the need for further mechanistic studies that describe which key cell populations are beneficially targeted (57, 58).

The role of ORMDL3 in sphingolipid regulation is also salient in light of mounting evidence linking sphingolipid metabolites and these diseases. Sphingosine-1-phosphate (S1P) signaling through its receptor S1PR is critical for leukocyte trafficking and cytokine-induced protein expression, both of which have been implicated in RA pathology (59). Indeed, both mouse models and human clinical trials have shown that inhibition of S1P results in decreased circulating lymphocytes and a therapeutic reduction of RA disease severity (60). Ceramide, a central sphingolipid metabolite, has also been linked to inflammatory disorders, particularly type 2 diabetes (61, 62). Type 2 diabetes patients display elevated plasma ceramide levels, and ceramide accumulation contributes to insulin resistance through activation of inflammatory cytokines such as TNF-α (61, 63). In fact, the conversion from sphingomyelin to ceramide by sphingomyelinases represents a potent pro-inflammatory signal in many cell types (64). Ceramide-1 phosphate also increases macrophage migration and inflammation (64). These are only a few examples which highlight the importance of maintaining sphingolipid homeostasis in the prevention of several inflammatory disorders. As ORMDL3 is a major regulator of SPT, the rate limiting enzyme that catalyzes all sphingolipid biosynthesis, we posit that increased ORMDL3 expression and dysregulation of sphingolipid levels likely exacerbates asthma through effects in multiple cell types, including T cells. Ample evidence indicates that variations in ORMDL3 expression directly affect levels of S1P and ceramides in various cells and tissues (25, 58, 65–67), sometimes in unpredictable ways. Changes in membrane sphingolipids may also simply disrupt TCR signaling *via* alterations in lipid raft composition. Moreover, a recent study from the Worgall group quantified sphingolipids in plasma and whole blood samples in children with or without asthma, linking 17q21 SNPs associated with elevated ORMDL3 expression to lower circulating sphingolipid species (e.g. ceramides) (66). Moreover, *in vitro* experiments demonstrated lower *de novo* sphingolipid synthesis in peripheral blood cells from children with asthma compared to controls. Considering T cells comprise 40–60% of blood leukocytes, subsequent studies should test whether T cells are indeed the major driver of altered circulating sphingolipids in asthmatic children carrying the 17q21 risk SNPs.

Defining the mechanisms by which altered ORMDL3 expression perturbs CD4^+^ T cell differentiation and function should illuminate new treatment paradigms for patients with 17q12–21 risk SNPs, and expand of our current understanding of asthma pathogenesis.

## Author Contributions

CL wrote the manuscript. TD edited the manuscript. AS constructed **Figure 1** and edited the manuscript. All authors contributed to the article and approved the submitted version.

## Funding

This work was supported with funding from the Collaborative Health Initiative Research Program (CHIRP) at USUHS.

## Conflict of Interest

The authors declare that the research was conducted in the absence of any commercial or financial relationships that could be construed as a potential conflict of interest.
